# CytoSorb^®^ Hemoadsorption in Post-Cardiac Arrest Syndrome After Out-of-Hospital Cardiac Arrest: A Propensity Score-Matched Cohort Study

**DOI:** 10.3390/biomedicines14040930

**Published:** 2026-04-19

**Authors:** Julian Kreutz, Klevis Mihali, Vivien Sievertsen, Lukas Harbaum, Georgios Chatzis, Styliani Syntila, Bernhard Schieffer, Birgit Markus

**Affiliations:** Department of Cardiology, Angiology, and Intensive Care Medicine, University Hospital, Philipps-Universität Marburg, Baldingerstrasse, 35043 Marburg, Germany

**Keywords:** out-of-hospital cardiac arrest, post-cardiac arrest syndrome, hemoadsorption, CytoSorb, ischemia–reperfusion injury

## Abstract

**Background**: Post-cardiac arrest syndrome (PCAS) following out-of-hospital cardiac arrest (OHCA) is driven by global ischemia–reperfusion injury, endothelial dysfunction, and a dysregulated inflammatory response. This cascade frequently culminates in profound vasoplegia and multiorgan failure, even when guideline-directed post-resuscitation management is applied. Hemoadsorption using the CytoSorb device may attenuate hyperinflammation and vasoplegia by removing circulating inflammatory and injury-related mediators. **Methods**: This single-centre, retrospective cohort study compared adults with PCAS following OHCA who received hemoadsorption with propensity score-matched controls (1:1 matching; *n* = 50 per group). For patients treated with hemoadsorption, data were analyzed within predefined intervals covering the 24 h preceding therapy initiation (T1) and the 24 h following the completion of the hemoadsorption treatment period (T2). Controls were evaluated at time points aligned to those of their matched hemoadsorption counterparts. Hemodynamic, metabolic, respiratory, and organ injury markers were assessed. **Results**: Formal between-group comparisons of temporal change between T1 and T2 showed no statistically significant differences between hemoadsorption-treated patients and matched controls across key parameters, including VIS (Δ −18.7 vs. −7.7; *p* = 0.183) and lactate (Δ −1.8 vs. −1.25 mmol/L; *p* = 0.780), as well as markers of organ injury, pH, and oxygenation. In exploratory ANCOVA models, only base excess was associated with treatment group (*p* = 0.035). Survival to hospital discharge was comparable (48% vs. 40%; *p* = 0.423), with similar neurological outcomes. Within the hemoadsorption group, pre–post comparisons around hemoadsorption initiation (T1–T2) demonstrated marked improvements, including reduced vasoactive support (VIS 70.0 to 12.1; *p* = 0.039), substantial lactate clearance (4.1 to 1.1 mmol/L; *p* < 0.001), and declines in organ injury markers (AST, ALT, LDH, myoglobin), alongside more pronounced platelet reduction compared with controls (129 to 57 × 10^3^/µL vs. 189 to 123 × 10^3^/µL). However, adjusted analyses indicated that these changes were primarily driven by baseline shock severity rather than a treatment-specific effect. **Conclusions**: In this propensity score-matched cohort of PCAS patients after OHCA, hemoadsorption was associated with within-group physiological changes but showed no detectable advantage over matched controls, with similar survival. These findings are hypothesis-generating and warrant prospective studies with standardized timing and phenotype-guided patient selection.

## 1. Introduction

Patients who achieve return of spontaneous circulation (ROSC) after out-of-hospital cardiac arrest (OHCA) frequently develop post-cardiac arrest syndrome (PCAS). This multisystemic disorder is caused by global ischaemia–reperfusion injury, which leads to immune–endothelial dysregulation and microcirculatory failure [1]. Contemporary international guidelines (ERC/ESICM, ILCOR, and AHA) recommend the use of structured post-resuscitation care bundles [2,3,4]. These include hemodynamic optimisation, targeted fever prevention/temperature control, lung-protective ventilation, seizure control, and early coronary evaluation when indicated. However, guidelines provide limited evidence-based strategies that directly address the systemic PCAS response. Despite adherence to these pathways, clinical outcomes remain suboptimal [5]. The pathobiology of PCAS features an early surge of danger-associated molecular patterns (DAMPs), complement activation, and cytokines (e.g., IL-6 and TNF-α), which lead to endothelial activation, glycocalyx shedding, capillary leak, and vasoplegia [6]. Consequently, microvascular flow becomes heterogeneous, impairing oxygen extraction and decoupling macrocirculatory targets from tissue perfusion, thereby promoting multiorgan dysfunction. 

Extracorporeal hemoadsorption using the CytoSorb device is based on highly porous, biocompatible polymer beads. The single-use cartridge can be incorporated into continuous renal replacement therapy (CRRT), veno-arterial extracorporeal membrane oxygenation (VA-ECMO), or standalone circuits. This configuration enables the removal of inflammatory mediators, myoglobin, bilirubin, and other circulating solutes involved in immune-endothelial activation, vasoplegia, and microcirculatory failure. In sepsis and other hyperinflammatory states, randomised trials and meta-analyses have demonstrated inconsistent effects on cytokine levels, reflecting variability in timing, patient selection, and disease severity [7,8]. In cardiogenic shock (CS), including cohorts supported by mechanical circulatory support (MCS), observational studies have reported reductions in the vasoactive–inotropic score (VIS), improved lactate kinetics, and decreases in organ injury markers [9]. There is limited and heterogeneous evidence for hemoadsorption in PCAS. The CYTER randomised trial by Supady et al. did not demonstrate improved clinical outcomes following cardiac arrest with CytoSorb therapy [10]. However, smaller observational studies and case series have reported reductions in inflammatory mediators, such as interleukin-6, following hemoadsorption, whereas other analyses have suggested unfavourable outcomes in selected patient groups treated with extracorporeal adsorption devices [11,12]. Ongoing registry initiatives, such as the international COSMOS study, aim to define the real-world utilisation, safety, and phenotype-specific efficacy of hemoadsorption, thereby guiding the timing and patient selection for future controlled trials [13].

This single-centre, retrospective, propensity score-matched cohort study investigates hemoadsorption as an adjunct to contemporary post-resuscitation care, analysing the short-term trajectories of hemodynamic, metabolic, and organ injury parameters surrounding therapy initiation.

## 2. Materials and Methods

### 2.1. Study Design and Setting

This retrospective, single-centre, observational cohort study was conducted by the Department of Cardiology, Angiology, and Intensive Care Medicine at the University Hospital of Marburg, Germany. Clinical, laboratory, and procedural data were extracted from the institutional electronic health records. 

### 2.2. Patient Population and Inclusion Criteria

All adult patients over the age of 18 who were admitted from January 2018 to December 2022 to the intensive care unit (ICU) at the University Hospital Marburg following an OHCA were screened retrospectively for eligibility. Patients who received hemoadsorption during post-cardiac arrest care were assigned to the CytoSorb group. Those who met the inclusion criteria but did not receive hemoadsorption were allocated to the control group. This group was retrospectively matched to the intervention cohort using predefined clinical, physiological, and laboratory variables to minimise confounding. All therapeutic decisions—including the initiation, timing, and duration of hemoadsorption, as well as the use of CRRT or temporary mechanical circulatory support (tMCS)—were at the full discretion of the attending physicians, following institutional protocols and current international post-resuscitation care guidelines. Patients received tMCS according to their clinical condition, using either a left ventricular micro-axial flow pump (mAFP; Impella CP, Abiomed, Danvers, MA, USA), VA-ECMO (CardioHelp, Getinge Group, Gothenburg, Sweden), or a combination of both systems (ECMELLA).

### 2.3. Intervention and Treatment Strategy

Hemoadsorption was implemented as an adjunctive extracorporeal therapy and either integrated into CRRT systems or used as a standalone procedure. Hemoadsorption in this study was performed using the CytoSorb cartridge (CytoSorbents Corporation, Princeton, NJ, USA), an extracorporeal adsorption device containing highly porous polymer beads designed to remove hydrophobic molecules with a molecular weight range of approximately 5–60 kDa. At the Department of Cardiology, Angiology, and Intensive Care Medicine at the University Hospital Marburg, hemoadsorption was introduced in 2018 as an adjunctive therapy for selected patients with severe PCAS characterized by vasoplegic shock, escalating vasopressor requirements, systemic inflammatory response, or evolving multiorgan dysfunction despite standard post-resuscitation care. The decision to initiate hemoadsorption was made by the treating intensivist based on the overall clinical condition and hemodynamic profile of the patient. In practice, typical triggers included increasing vasopressor demand, persistent metabolic acidosis or hyperlactatemia, and progressive organ dysfunction despite guideline-directed post-resuscitation management. In most cases, hemoadsorption was integrated into an already established CRRT circuit. Treatment sessions were maintained for a minimum of 24 h, with daily multidisciplinary reassessment of the indication and hemodynamic response. To ensure optimal adsorption efficiency, adsorber cartridges were replaced every 12–24 h, according to manufacturer recommendations and institutional protocols. Systemic anticoagulation during hemoadsorption was managed according to unit protocol using unfractionated heparin, titrated to achieve a target activated partial thromboplastin time (aPTT) of approximately 50 to 60 s.

### 2.4. Propensity Score Matching

To reduce selection bias and ensure comparability between treatment groups, a propensity score matching (PSM) approach was implemented. Propensity scores were calculated using a multivariable logistic regression model that included clinically relevant pre-treatment covariates likely to affect both the probability of receiving hemoadsorption therapy and clinical outcomes. The model incorporated the following variables: age, duration of CPR until ROSC, admission arterial pH, admission arterial lactate concentration, use of MCS during the hospitalization, application of targeted temperature management (TTM), and performance of coronary angiography. Hemoadsorption-treated patients were matched to control patients who did not receive hemoadsorption in a 1:1 ratio using nearest-neighbor matching without replacement and a caliper of 0.2 standard deviations of the logit-transformed propensity score. The performance of the propensity score model was evaluated using the C-statistic and the Hosmer–Lemeshow test was used to assess calibration. The balance of covariates before and after matching was assessed using standardised mean differences (SMDs).

### 2.5. Data Collection and Outcomes

Baseline time (T0) was defined as the time of admission to the ICU. For patients receiving hemoadsorption, two additional predefined time intervals were established to assess within-patient trajectories: T1, the 24 h interval preceding the initiation of hemoadsorption, and T2, the 24 h period following completion of hemoadsorption treatment. The observation windows were aligned with the initiation of hemoadsorption to allow assessment of short-term physiological responses to therapy rather than the entire temporal course following cardiac arrest. To ensure robust comparability between groups, data for the control group (non-hemoadsorption group) were obtained at time points aligned with the treatment trajectory of their matched hemoadsorption counterparts. This temporal alignment enabled a parallel assessment of hemodynamic, metabolic, and organ function parameters in both cohorts. An exploratory subgroup analysis was performed comparing early (<48 h) versus late (≥48 h) initiation of hemoadsorption.

Collected variables at T0, T1, and T2 included demographic characteristics, pre-existing comorbidities, and cardiac arrest-related parameters. Baseline severity was evaluated using standard indices of physiological and organ dysfunction. Organ support parameters included TTM, ventilation settings, CRRT, and the need for tMCS. Laboratory assessments included inflammatory, metabolic, hepatic, renal and haematological parameters, which were obtained as part of routine intensive care monitoring. Neuron-specific enolase (NSE) concentrations were measured for neurological prognostication following cardiac arrest, with samples obtained at approximately 24, 48 and 72 h after ROSC, in accordance with institutional protocols. Cytokine measurements, such as those of IL-6 or TNF-α, were not routinely performed during the study period and were therefore not systematically available for analysis.

T1–T2 analyses were performed using an available-case approach, including only patients with paired measurements at both timepoints. Patients who died before reaching T2 did not contribute follow-up data and were therefore excluded from longitudinal analyses. To ensure comparability between groups, analyses were restricted to matched pairs in which both patients were alive at the corresponding timepoints. As follow-up availability varied across variables, effective sample sizes differed between parameters, and only available observations were included for each analysis.

### 2.6. Statistical Analysis

All statistical computations were carried out using SPSS Statistics (version 29; IBM Corp., Armonk, NY, USA) and GraphPad Prism (version 10; GraphPad Software, San Diego, CA, USA). The distribution of continuous variables was assessed by the Shapiro–Wilk test. Variables with approximately normal distribution were summarized as mean ± standard deviation (SD), whereas skewed data were expressed as median values with corresponding interquartile ranges (IQR). Between-group differences were analysed using independent-sample *t*-tests or the Mann–Whitney U–test, depending on the data distribution. For comparisons within a patient or within a pair, paired *t*-tests or Wilcoxon signed-rank tests were used as appropriate. Categorical variables were compared using the χ^2^ test or Fisher’s exact test when expected frequencies were small. Effect sizes were reported where relevant to aid interpretation beyond nominal significance. All analyses were two-tailed, and a *p*-value < 0.05 was considered statistically significant. Standardized mean differences (SMDs) were used to evaluate covariate balance between the matched groups, with Cohen’s d reported for continuous variables where appropriate. Formal between-group comparisons of temporal change between T1 and T2 were performed using unadjusted analyses of change scores and exploratory analysis of covariance (ANCOVA) models. For the unadjusted comparisons, the Mann–Whitney U test was used to compare T2–T1 change scores between groups. For ANCOVA, follow-up values at T2 were modelled as dependent variables, with treatment group as the factor and the corresponding baseline value at T1 as the covariate. Exploratory multivariable linear regression analyses were performed to evaluate the independent association between hemoadsorption and changes in key physiological parameters. The models included CytoSorb therapy, baseline lactate, baseline VIS and CRRT use as covariates. In an exploratory analysis, VIS was assessed at 24 and 72 h after the initiation of CytoSorb therapy and compared using the Friedman test. As follow-up availability varied across variables, T1–T2 analyses were performed on an available-case basis.

### 2.7. Ethical Approval and Consent to Participate

The study protocol was approved by the local ethics committee of the Faculty of Medicine, Philipps-University Marburg (ethics reference number: 24-12 RS). All procedures were conducted in accordance with the Declaration of Helsinki and relevant national regulations. Due to the retrospective nature of the study, the requirement for written informed consent was waived by the ethics committee.

## 3. Results

### 3.1. Overview of the Study Cohort

**Figure 1:**
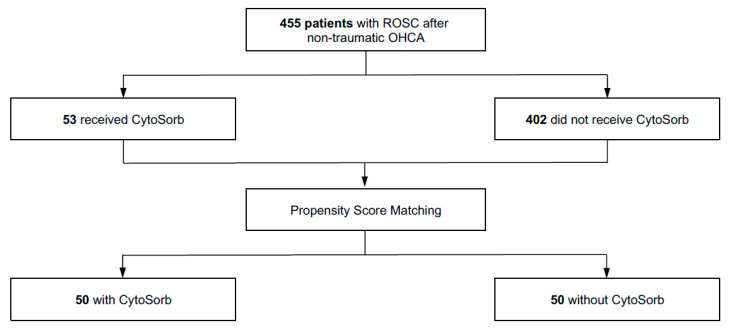
Flowchart of the study cohort.

During the study period, 455 adults who met the inclusion criteria following non-traumatic OHCA were admitted to the ICU. Of this cohort, 53 patients (11.6%) received hemoadsorption during ICU therapy, while 402 patients (88.4%) did not. Following 1:1 nearest-neighbour PSM without replacement, 50 CytoSorb-treated patients were successfully paired with 50 closely matched controls (see Figure 1).

### 3.2. Characteristics of the Unmatched and the PSM Cohorts

**Figure 2:**
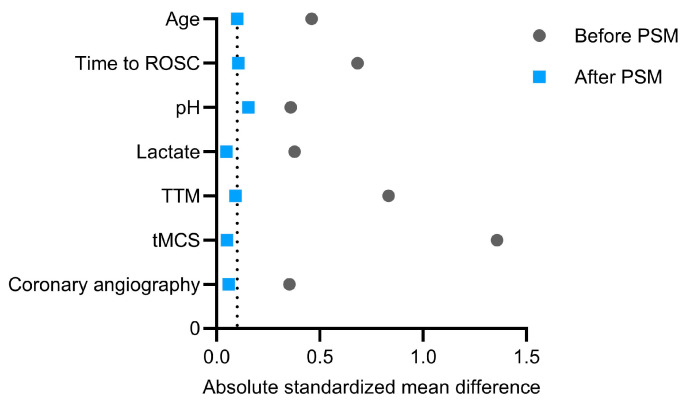
Love plot of absolute standardized mean differences for the seven matching variables before and after propensity score matching; dashed line indicates the 0.1 threshold.

**Figure 3:**
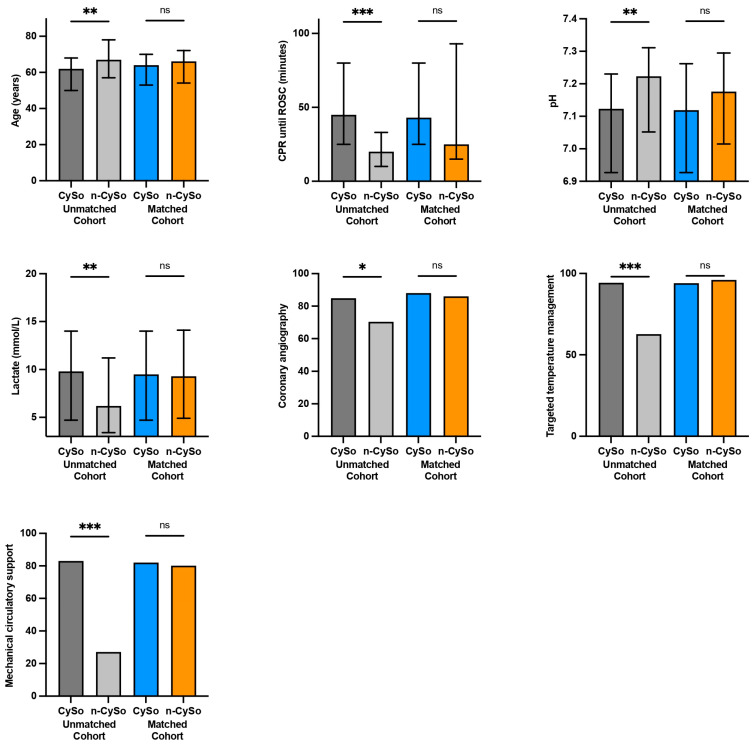
Comparison of key matching variables between patients treated with CytoSorb (CySo) and without CytoSorb (n-CySo) before and after 1:1 matching: * *p* < 0.05, ** *p* < 0.01, *** *p* < 0.001, (ns: not significant).

In the unmatched cohort, hemoadsorption-treated patients presented with a noticeably more compromised clinical profile at ICU admission. PSM substantially improved covariate balance across the seven prespecified matching variables (Figure 2 and Figure 3). The propensity score model showed modest discrimination, with a C-statistic of 0.621 (95% CI, 0.508–0.734; *p* = 0.037), and acceptable calibration (Hosmer–Lemeshow *p* = 0.092). Standardized mean differences after matching were 0.100 for age, 0.106 for time to ROSC, 0.154 for pH, 0.048 for lactate, 0.092 for targeted temperature management, 0.051 for tMCS, and 0.059 for coronary angiography, indicating improved overall balance, although mild residual imbalance remained for pH and time to ROSC.

The presumed causes of cardiac arrest were distributed similarly between the groups before and after matching. In the matched cohort, cardiac aetiology was the most frequent cause, occurring slightly more frequently in the control group than in the hemoadsorption group (66% vs. 58%; *p* = 0.412), while non-cardiac causes occurred at comparable rates. Determining the definitive cause of arrest is often challenging in critically ill post-resuscitation patients; therefore, some cases remained classified as non-cardiac or of uncertain origin.

### 3.3. Baseline Values (T0), Course of Hospitalization, and Outcome

Upon admission to the ICU (T0), patients in the hemoadsorption group exhibited less favourable baseline hemodynamic and organ function parameters compared to the matched controls. They required higher vasoactive–inotropic support (VIS: 64.22 [IQR 21.21–138.67] vs. 23.53 [IQR 14.29–125]; *p* = 0.054), had a modestly higher SOFA score (12 [IQR 11–13] vs. 11 [IQR 10–12]; *p* = 0.005), and had a lower PaO_2_/FiO_2_ ratio (137 [IQR 101–340] vs. 228 [IQR 160–314]; *p* = 0.151). At T0, the median serum creatinine level was 1.47 mg/dL (IQR 1.2–1.8) in the hemoadsorption group, compared to 1.25 mg/dL (IQR 1.0–1.7) in the control group. This reflects a modest, yet statistically significant, difference in baseline renal function between the two groups (*p* = 0.027). These residual differences likely reflect confounding by indication, as hemoadsorption was preferentially initiated in patients with more pronounced circulatory instability despite the use of propensity score matching.

Table 1 summarizes demographics, comorbidities, cardiac arrest characteristics, initial blood gas analysis, coronary angiography, and mechanical circulatory support in the overall and matched cohorts.

Hemoadsorption cartridges were integrated into CRRT in 48 of 50 patients (96%), while it was used as a standalone procedure in the remaining two patients. In the matched cohort, 17 of 50 patients (34%) in the control group also received CRRT during ICU stay, including during a comparable observation interval aligned to their matched counterparts. In most patients, CRRT had already been initiated prior to hemoadsorption and remained ongoing throughout the T1–T2 observation period. The median total RRT duration was 103 h (IQR 41–240) for hemoadsorption-treated patients and 117 h (IQR 27–198) for the control group (*p* = 0.852). Patients treated with hemoadsorption received a median of 3.0 adsorber cartridges (IQR 1.0–4.0). The initiation of hemoadsorption occurred at a median of 42.5 h (IQR 14.5–123.5) after initial hospital admission. This timing reflects the clinical use of hemoadsorption as a rescue or escalation therapy in patients with evolving circulatory instability rather than as an early anti-inflammatory intervention immediately after ROSC. The median duration of hemoadsorption treatment was 72.0 h (IQR 24.0–72.0).

To further address potential confounding by concomitant CRRT, we performed an exploratory subgroup analysis restricted to patients receiving CRRT in both groups (hemoadsorption: *n* = 48; controls: *n* = 17). In this subgroup, no statistically significant between-group differences were observed for changes in lactate, VIS, LDH, or myoglobin. Median Δlactate was −1.8 mmol/L in the hemoadsorption group versus −0.6 mmol/L in the controls (Mann–Whitney U = 186.0, *p* = 0.919). Median ΔVIS was −18.7 versus −0.3 (U = 76.0, *p* = 0.160), median ΔLDH was −228 versus 17 (U = 149.0, *p* = 0.298), and median Δmyoglobin was −688 versus 211 (U = 115.0, *p* = 0.284). Although the numerical direction of change remained favorable for the hemoadsorption group for several variables, no between-group difference was detectable in this CRRT-restricted analysis. In particular, Δlactate showed no signal of a treatment-related difference, whereas the interpretation of the remaining endpoints should be cautious given the reduced subgroup size and the limited availability of paired follow-up measurements, particularly among CRRT-treated controls.

**Figure 4:**
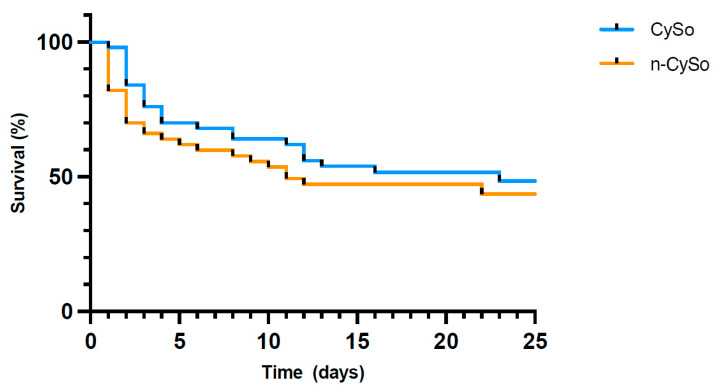
Kaplan–Meier curves for in-hospital survival in the propensity score-matched cohorts with CytoSorb (CySo) and without CytoSorb (n-CySo), (*p* = 0.423).

Hemoadsorption-treated patients generally presented with less favourable overall clinical conditions at ICU admission, but they had a numerically higher survival rate at hospital discharge than the matched cohort (48% vs. 40%), without reaching statistical significance (*p* = 0.423) (Figure 4). Among patients in the hemoadsorption group who died during hospitalization, 13 deaths occurred during ongoing hemoadsorption treatment, whereas another 13 patients died after termination of hemoadsorption therapy. Causes of death were not systematically adjudicated; however, chart review suggested that most deaths were related to hypoxic–ischemic brain injury or progressive multiorgan failure rather than isolated refractory circulatory collapse.

At discharge, the neurological outcomes of the hemoadsorption and control groups were comparable, as assessed by the CPC score. Thirteen of the 24 survivors (54%) in the hemoadsorption group achieved a CPC of 1–2, compared with 11 of the 20 survivors (55%) in the control group (*p* = 1.00). This alignment in functional neurological status was reflected biochemically by similar peak concentrations of NSE. In the matched cohort, the median NSE level was 78 µg/L (IQR 49–170) in the hemoadsorption group and 84 µg/L (IQR 48–125) in the control group, with no statistically significant difference (*p* = 0.827). The median length of hospital stay showed a non-significant trend toward longer hospitalization among hemoadsorption-treated patients (13 days [IQR 3–23] vs. 9 days [IQR 1–19]; *p* = 0.070).

### 3.4. Comparison of Periods T1 and T2

During the T1–T2 observation period, 13 patients in the hemoadsorption group and 16 in the matched control group died before reaching T2 and were therefore not included in T1–T2 analyses. To ensure comparability, analyses were restricted to matched pairs in which both patients were alive at the corresponding timepoints, resulting in 25 matched pairs with complete data at T1 and T2.

At the T1 timepoint, patients in the hemoadsorption group exhibited higher levels of inflammatory and tissue injury markers, consistent with the clinical indication for hemoadsorption in patients with more pronounced systemic inflammation and organ dysfunction. In the hemoadsorption group, inflammatory markers increased between T1 and T2, with CRP rising from 102.8 to 193.0 mg/L (*p* = 0.012) and PCT from 1.4 to 2.1 ng/mL (*p* = 0.035). Platelet counts declined markedly during hemoadsorption therapy (129 × 10^3^/µL to 57 × 10^3^/µL; *p* < 0.001), representing a substantially greater reduction than in matched controls (189 × 10^3^/µL to 123 × 10^3^/µL; *p* = 0.040).

Significant reductions in organ injury markers—including AST (*p* = 0.036), ALT (*p* = 0.010), LDH (*p* = 0.039), and myoglobin (*p* = 0.012)—were observed only in the hemoadsorption cohort, whereas corresponding changes remained non-significant in the control group. Baseline myoglobin concentrations were higher in the hemoadsorption group at T1, consistent with the clinical indication for hemoadsorption in patients with more pronounced tissue injury. Within-group analyses suggested metabolic improvement in the hemoadsorption cohort, as base excess increased from −2.9 to 1.8 mmol/L (*p* = 0.008) and lactate declined from 4.1 to 1.1 mmol/L (*p* < 0.001), compared with a smaller reduction in matched controls (1.5 to 1.0 mmol/L; *p* = 0.004). Exploratory multivariable regression analyses identified baseline lactate as the strongest predictor of lactate reduction (B −0.775, *p* < 0.001). Baseline VIS was also independently associated with lactate change (*p* = 0.045), whereas neither hemoadsorption therapy (*p* = 0.922) nor CRRT use (*p* = 0.749) were independently associated with Δlactate after adjustment for baseline severity parameters. Consistent findings were observed in ANCOVA models adjusting for baseline lactate, VIS, and CRRT use. These analyses suggest that the magnitude of metabolic improvement was primarily related to baseline shock severity rather than to hemoadsorption itself.

Table 2 summarises formal between-group comparisons of temporal changes. Unadjusted analyses of change scores revealed no statistically significant differences between groups across the investigated variables. In ANCOVA models adjusting for baseline values, follow-up base excess was the only variable associated with treatment group, with no significant treatment-group effects observed for the remaining variables. These findings suggest that most between-group differences in temporal change could not be statistically confirmed in this relatively small cohort.

Exploratory subgroup analyses comparing early (<48 h; *n* = 28) versus late (≥48 h; *n* = 22) initiation of hemoadsorption demonstrated a greater reduction in lactate with earlier initiation (median Δlactate −3.45 mmol/L [IQR −7.60 to −1.70; *n* = 18/28] vs. −0.30 mmol/L [IQR −1.20 to 0.10; *n* = 21/22]; *p* = 0.017). ΔVIS did not differ between subgroups (*p* = 0.703). Additional exploratory differences were observed for Δcreatine kinase (*p* = 0.009), Δplatelets (*p* < 0.001), ΔCRP (*p* = 0.002), and ΔPCT (*p* = 0.006), whereas no consistent differences were seen across the remaining parameters.

In 26 hemoadsorption-treated patients with VIS data available at all three time points, vasoactive requirements declined significantly over time, with median values decreasing from 53.4 before CytoSorb to 8.2 at 24 h and 5.0 at 72 h after initiation (Friedman χ^2^ = 27.327, *p* < 0.001). Post hoc Wilcoxon tests confirmed significant reductions from baseline to 24 h, from baseline to 72 h, and from 24 to 72 h (all *p* ≤ 0.003).

Respiratory parameters remained broadly stable over time in both groups (Table 3). In the hemoadsorption cohort, ventilatory settings, including PEEP (10 to 9 mbar; *p* = 0.574) and peak inspiratory pressure (26 to 24 mbar; *p* = 0.749) showed no significant change between T1 and T2. The PaO_2_/FiO_2_ ratio exhibited a modest, non-significant improvement (160 to 195; *p* = 0.530). In contrast, matched controls demonstrated a slight numerical decline in oxygenation (249 to 218; *p* = 0.469) despite unchanged ventilatory settings.

Overall, the descriptive within-group changes between T1 and T2 were more pronounced in the hemoadsorption cohort. However, these findings should be interpreted considering the observational design and the fact that there were no significant differences between the groups.

## 4. Discussion

PCAS is characterized by an intense, rapidly evolving, ischemia–reperfusion-driven inflammatory surge that contributes to profound vasoplegia, microcirculatory failure, and early multiorgan dysfunction [14,15]. The clinical variability reflects the heterogeneous nature of the condition. Despite advances in post-resuscitation care, effective modulation of this hyperacute inflammatory phase remains an unmet clinical need. Stabilizing hemodynamics and preventing progressive organ injury during this phase may be essential to improve outcomes [16,17]. This PSM analysis of patients with PCAS following OHCA revealed that hemoadsorption was not associated with a detectable treatment effect in direct between-group comparisons of temporal change, and that survival was similar between groups. Although improvements in hemodynamic and metabolic parameters were observed within groups, these findings must be interpreted cautiously as they likely reflect overall clinical stabilisation rather than a treatment-specific effect. In particular, the frequent use of extracorporeal organ support alongside other treatments may have substantially influenced these trajectories. CRRT can improve metabolic parameters by correcting acid–base disturbances and fluid balance. Meanwhile, VA-ECMO and adjustments in extracorporeal flow may affect tissue perfusion, oxygen delivery and vasopressor requirements. Furthermore, vasoactive therapy was titrated dynamically according to clinical need. Taken together, these factors limit causal inference, and the observed changes should be considered associative and hypothesis-generating.

Although the present data did not demonstrate a detectable treatment-specific effect in direct between-group analyses, reductions in VA-ECMO flow requirements, lower VIS, and improved lactate kinetics are compatible with mechanistic concepts suggesting that extracorporeal mediator modulation may attenuate vasoplegia and improve microcirculatory coherence [13,18]. From a pathophysiological perspective, these observations are biologically plausible as ischemia–reperfusion triggers an exaggerated release of cytokines, DAMPs, and vasoactive mediators that, in summary, promote nitric-oxide-mediated vasodilation, endothelial barrier disruption, mitochondrial dysfunction, and impaired catecholamine responsiveness [19,20]. Previous studies on vasoplegic shock and CS have also demonstrated that hemoadsorption has been associated with reductions in cytokines and other circulating mediators involved in capillary leak, endothelial dysfunction, and impaired tissue perfusion [21,22]. The reductions in organ injury markers (AST, ALT, LDH, and myoglobin) observed in the hemoadsorption group may reflect a combination of improved hemodynamic stabilization, recovery from ischemia–reperfusion injury, and the potential removal of circulating injury-related mediators, but alternative explanations related to concomitant therapies and overall shock recovery remain equally possible. These findings should be interpreted in the context of an inconclusive and heterogeneous evidence base for haemoperfusion in cardiac arrest patients. While the randomised CYTER trial did not demonstrate improved clinical outcomes, observational data have reported both neutral and adverse associations, alongside small studies suggesting reductions in inflammatory mediators such as interleukin-6. This variability highlights the biological heterogeneity of PCAS and suggests that any potential benefit may depend on patient selection, timing and inflammatory phenotype. In line with this, our exploratory subgroup analysis indicated greater lactate reduction with earlier initiation (within 48 h), without a corresponding effect on VIS. However, these findings should be interpreted with caution given the exploratory design, variable-specific missingness and lack of adjustment for multiple testing. Notably, adjusted analyses consistently showed that changes in lactate and hemodynamic support were primarily driven by baseline shock severity rather than hemoadsorption, suggesting regression to the mean in patients with more pronounced initial derangement.

Although CRP and PCT increased between T1 and T2 in both cohorts, these trends are consistent with the natural trajectory of the PCAS rather than a treatment-specific effect. As delayed acute-phase reactants, CRP and PCT primarily reflect hepatic transcriptional activation occurring several hours after the initial cytokine release [23,24]. Accordingly, these parameters do not capture the early mediator-driven phase targeted by hemoadsorption. Changes in lactate, base excess, organ injury markers, and VA-ECMO flow should therefore be interpreted cautiously, as they cannot be attributed to mediator modulation based on the present data. Base excess was the only parameter associated with treatment group in exploratory ANCOVA models and should be interpreted cautiously given multiple testing. Similar dissociations between early clinical stabilization and inflammatory marker kinetics have been described in sepsis and CS [25,26].

Safety considerations are particularly relevant in patients requiring multiple extracorporeal circuits. The more pronounced decline in platelet counts observed during hemoadsorption in our cohort is consistent with prior reports from critically ill populations, indicating adsorption- or activation-related thrombocytopenia [27]. Mechanistically, platelets may adhere to or become activated by the polymer matrix, while nonspecific binding of albumin and other plasma proteins contributes to reduced serum levels. Importantly, the clinical relevance of the observed platelet decline remains uncertain, as bleeding events, transfusion requirements, and systematic assessment for heparin-induced thrombocytopenia (HIT) were not captured in the present dataset. Systematic monitoring of platelet counts and albumin levels—and proactive substitution strategies—remain essential when integrating hemoadsorption into complex post-cardiac arrest care.

Despite the potential physiological benefits, hemoadsorption therapy did not improve survival or neurological outcomes in the matched cohort. This disconnect likely reflects the overarching impact of irreversible hypoxic–ischemic brain injury and delayed multiorgan failure, dominant determinants of late mortality that may not be modifiable once established. The timing of intervention emerges as a critical consideration: hemoadsorption initiated after substantial neuronal or microcirculatory injury may be unable to influence ultimate neurological recovery [28,29]. Furthermore, PCAS is a heterogeneous syndrome, and it is plausible that only a subset of patients with a “high-inflammatory” or “shock-dominant” phenotype derive meaningful benefit from early mediator modulation [30].

Nevertheless, the observed short-term changes across hemodynamic, metabolic, and biochemical parameters could support the biological plausibility of hemoadsorption as an adjunctive therapy in selected patients with PCAS, while also emphasizing the dominant influence of baseline shock severity on early recovery trajectories. However, given the observational design of the study and the frequent use of concomitant extracorporeal organ support modalities, including CRRT and mechanical circulatory support, these findings must be interpreted cautiously and should be regarded as hypothesis-generating rather than indicative of a causal treatment effect. Prospective studies with standardized treatment timing, clearly defined inclusion criteria, and biomarker-guided patient selection are needed to determine whether mediator-targeted hemoadsorption can translate into clinically meaningful outcome improvements in PCAS.

## 5. Limitations

Several limitations must be acknowledged when interpreting these findings. The retrospective single-centre design introduces a risk of selection bias. PSM was performed using clinically relevant baseline variables to address this issue; however, residual confounding may persist. In particular, the propensity score model showed only modest discrimination, and clinically relevant differences in baseline severity remained after matching, indicating that PSM reduced but did not eliminate confounding by indication. Notably, hemoadsorption was initiated more frequently in patients with greater circulatory instability and systemic inflammation, reflecting the confounding by indication commonly observed in observational studies of critically ill populations. The matched cohort was relatively small, potentially limiting the statistical power to detect moderate differences in clinical outcomes such as survival or neurological recovery. The neurological outcome was assessed using the CPC at hospital discharge. This represents an early and relatively broad endpoint, which may not reflect long-term neurological recovery. Additionally, treatment decisions regarding the initiation, timing, and duration of hemoadsorption were made at the discretion of treating physicians, based on the patient’s condition. The broad variability in the timing of hemoadsorption initiation reflects heterogeneous real-world use as an individualized rescue or escalation strategy rather than a standardized protocol-driven intervention, which limits comparability between patients and should be considered when interpreting the findings and their generalizability. As a result, therapy initiation was not standardized and often occurred late, which may have limited the assessment of potential benefits associated with earlier mediator removal during the early phase of PCAS. Mortality before T2 introduced informative censoring, as 13 hemoadsorption-treated patients and 16 controls died before follow-up and were not included in T1–T2 analyses. To ensure comparability, analyses were restricted to matched pairs in which both patients were alive at the time of assessment, resulting in 25 pairs. While this survivor-based restriction may have biased the observed trajectories, the direction of this bias remains uncertain; however, this approach was necessary to allow a valid comparison of temporal changes and to assess potential treatment-related effects of hemoadsorption. Consequently, effective sample sizes were reduced and variable-specific, with additional missingness for selected parameters. In addition, the revised early (<48 h) versus late (≥48 h) subgroup analysis involved multiple unadjusted comparisons across several parameters; therefore, the nominally significant findings observed for selected variables should be interpreted cautiously and considered hypothesis-generating.

Hemoadsorption was often implemented alongside ongoing extracorporeal organ support, most commonly within CRRT. Because hemoadsorption was applied alongside CRRT, VA-ECMO, and dynamically adjusted catecholamine therapy in many patients, the specific contribution of the adsorber to the observed physiological changes cannot be reliably separated from the effects of concurrent supportive interventions. Cytokine concentrations were not routinely measured during the study period. Although this approach reflects real-world clinical practice in many centers, it limits mechanistic understanding of the extent of inflammatory mediator removal achieved by hemoadsorption. The predefined sampling intervals may also fail to capture the rapid temporal dynamics of inflammatory mediators following cardiac arrest. Additionally, causes of death were not systematically adjudicated, which precludes detailed differentiation between neurological withdrawal of care, progressive multiorgan failure, or refractory circulatory shock.

Nevertheless, the study provides clinically relevant real-world data on short-term physiological trajectories associated with hemoadsorption in patients with severe PCAS.

## 6. Conclusions

In this propensity score-matched cohort of patients with PCAS following OHCA, hemoadsorption with CytoSorb was associated with within-group short-term changes in metabolic and hemodynamic parameters, but direct between-group analyses did not demonstrate a detectable advantage over matched controls. The observed physiological trajectories appeared to be strongly influenced by baseline shock severity and concurrent organ support. No survival or neurological benefit was observed. These findings support a cautious, hypothesis-generating interpretation and underscore the need for prospective controlled studies with standardized treatment timing and phenotype-guided patient selection.

## Figures and Tables

**Table 1:** Overview of demographics and comorbidities, cardiac arrest characteristics, initial blood gas analysis, coronary angiography and mechanical circulatory support in the overall and matched cohorts. Abbreviations: BMI: body mass index; CAD: coronary artery disease; MI: myocardial infarction; py: pack-years; COPD: chronic obstructive pulmonary disease; STEMI: ST-segment elevation myocardial infarction; CPR: cardiopulmonary resuscitation; ROSC: return of spontaneous circulation; VA-ECMO: veno-arterial extracorporeal membrane oxygenation; ECMELLA: VA-ECMO plus Impella; MCS: mechanical circulatory support. ^1^ n (%), ^2^ median (IQR).

	Unmatched Cohort			Matched Cohort		
Variable	CytoSorb (*n* = 53)	Non-CytoSorb (*n* = 402)	*p*-Value	CytoSorb (*n* = 50)	Non-CytoSorb (*n* = 50)	*p*-Value
**Demographics and comorbidities**						
Age (years) ^2^	62.0 (50–68)	67.0 (57–78)	0.002	64.0 (53–70)	66.0 (54–72)	0.400
Male sex ^1^	42 (79.2)	291 (72.4)	0.290	40 (80)	39 (78)	0.807
BMI (kg/m^2^) ^2^	28.1 (24.7–31.9)	27.8 (24.7–30.9)	0.229	28.0 (24.8–31.3)	26.3 (24.7–30.5)	0.218
Previous MI or known CAD ^1^	9 (17.6)	78 (19.5)	0.757	9 (18.8)	9 (20.5)	0.838
Arterial hypertension ^1^	31 (58.8)	204 (50.7)	0.275	30 (62.5)	17 (38.6)	0.039
Atrial Fibrillation ^1^	5 (9.8)	50 (12.5)	0.577	12 (25)	2 (4.5)	0.007
Heart Failure ≥ NYHA 3 ^1^	4 (7.8)	25 (6.1)	0.549	4 (8.3)	2 (4.5)	0.679
Pre-existing moderate/severe aortic or mitral valvular disease ^1^	2 (3.9)	32 (8.0)	0.404	4 (8.3)	5 (11.4)	0.732
Nicotine abuse (>5 py) ^1^	14 (25.5)	98 (24.5)	0.882	13 (27.1)	12 (27.3)	0.984
Alcohol Consumption ^1^	3 (5.9)	26 (6.4)	1.000	2 (4.2)	6 (13.6)	0.146
Diabetes mellitus ^1^	4 (7.8)	79 (19.7)	0.040	6 (12.5)	6 (13.6)	0.872
Chronic renal replacement therapy (RRT) ^1^	0 (0)	14 (3.5)	0.386	0 (0)	1 (2)	1.000
COPD ≥ GOLD 2 ^1^	2 (3.9)	40 (9.9)	0.204	2 (4.2)	2 (4.5)	1.000
Asthma ^1^	2 (3.9)	10 (2.4)	0.629	1 (2.1)	1 (2.3)	1.000
Stroke ^1^	2 (3.9)	37 (9.1)	0.288	5 (10.4)	3 (6.8)	0.716
Malignant disease ^1^	3 (5.9)	35 (8.8)	0.601	4 (8.3)	2 (4.5)	0.679
**Cardiac arrest characteristics**						
Witnessed arrest ^1^	38 (71.7)	282 (70.1)	0.817	35 (70)	35 (70)	1.000
Bystander CPR ^1^	33 (62.3)	235 (58.5)	0.597	32 (64)	32 (64)	1.000
Initial rhythm—shockable ^1^	28 (52.8)	168 (41.8)	0.128	28 (56)	26 (52)	0.690
Initial rhythm—asystole ^1^	11 (20.8)	125 (31.1)	0.123	10 (20)	11 (22)	0.807
Initial rhythm—pulseless electrical activity ^1^	14 (26.4)	14 (27.1)	0.914	12 (24)	13 (26)	0.818
CPR until ROSC ^2^	45 (25–80)	20 (10–33)	<0.001	43 (25–80)	25 (15–93)	0.170
Mechanical CPR ^1^	26 (49.1)	76 (19.0)	<0.001	23 (46)	19 (38)	0.420
**Initial parameters of blood gas analysis (at admission)**						
Lactate [mmol/L] ^2^	9.8 (4.7–14)	6.2 (3.4–11.2)	0.005	9.5 (4.7–14)	9.3 (4.9–14.1)	0.907
pH ^2^	7.12 (6.93–7.23)	7.22 (7.05–7.31)	0.005	7.12 (6.93–7.26)	7.176 (7.02–7.30)	0.282
**Coronary angiography**						
STEMI at admission ^1^	13 (24.5)	70 (17.4)	0.208	13 (26)	16 (32)	0.511
Diagnostic cardiac catheterization during in-hospital stay ^1^	45 (84.9)	283 (70.4)	0.027	44 (88)	43 (86)	0.767
Percutaneous coronary intervention ^1^	28 (52.8)	166 (41.3)	0.111	28 (56)	29 (58)	0.841
**Mechanical circulatory support**						
tMCS during hospitalization ^1^	44 (83.0)	109 (27.1)	<0.001	41 (82)	40 (80)	0.800
eCPR ^1^	20 (37.3)	53 (13.2)	<0.001	17 (34)	15 (30)	0.670
VA-ECMO ^1^	24 (45.3)	59 (14.7)	<0.001	21 (42)	19 (38)	0.685
Impella ^1^	7 (13.2)	33 (8.2)	0.297	7 (14)	16 (32)	0.033
ECMELLA ^1^	13 (24.5)	17 (4.2)	<0.001	13 (26)	5 (10)	0.038
**ICU procedures**						
Targeted temperature management ^1^	50 (94.3)	252 (62.7)	<0.001	47 (94.0)	48 (96.0)	1.000
Renal replacement therapy (RRT) ^1^	51 (96.2)	81 (20.1)	<0.001	48 (96.0)	17 (34.0)	<0.001
Total time of RRT in ICU (hours) ^2^	99 (35–247)	79 (23–153)	0.152	103 (41–240)	117 (27–198)	0.852
Duration of invasive ventilation (hours) ^2^	264 (60–388)	123 (12–224)	<0.001	266 (60–388)	188 (22–357)	0.073

**Table 2:** Between-group comparisons of temporal changes between T1 and T2 in the propensity score-matched cohorts. Abbreviations: VIS: vasoactive inotropic score; AST: aspartate aminotransferase; ALT: alanine aminotransferase; LDH: lactate dehydrogenase.

Variable	Non-CytoSorb Δ, Median (IQR)	CytoSorb Δ, Median (IQR)	Mann– Whitney U	*p*-Value	ANCOVA F	ANCOVA *p*-Value	Partial η^2^
VIS	−7.7 (−17.0 to 11.5)	−18.7 (−65.8 to −1.0)	165.0	0.183	0.035	0.853	0.001
Lactate	−1.25 (−4.6 to −0.3)	−1.8 (−6.4 to −0.2)	391.5	0.780	1.660	0.203	0.028
AST	−10.5 (−328.5 to 34.5)	−86.0 (−334.0 to 16.0)	389.5	0.453	0.522	0.473	0.009
ALT	−25.0 (−93.0 to −5.0)	−36.0 (−157.0 to 3.0)	371.5	0.311	0.151	0.699	0.003
LDH	−11.0 (−351.0 to 239.0)	−304.0 (−732.0 to 17.0)	333.0	0.236	0.198	0.658	0.003
Base excess	3.15 (0.5 to 7.55)	4.1 (1.7 to 7.1)	317.0	0.247	4.678	0.035	0.078
pH	0.045 (−0.07 to 0.12)	0.05 (−0.02 to 0.10)	348.0	0.512	1.582	0.214	0.028
Horovitz index	−31.0 (−190.5 to 13.5)	22.0 (−71.0 to 91.0)	282.5	0.292	0.311	0.579	0.006
Myoglobin	−173.0 (−972.0 to 546.5)	−527.0 (−2004.0 to −70.0)	260.5	0.120	1.124	0.294	0.020

**Table 3:** Temporal changes in laboratory, respiratory, and organ-support parameters before (T1) and after (T2) hemoadsorption period in the propensity score-matched cohorts. Abbreviations: CRP: C-reactive protein; PCT: procalcitonin; AST: aspartate aminotransferase; ALT: alanine aminotransferase; LDH: lactate dehydrogenase; mAFP: micro-axial flow pump; PEEP: positive end-expiratory pressure; pInsp: peak inspiratory pressure. ^2^ median (IQR).

	CytoSorb			non-CytoSorb		
	T1	T2	*p*-Value	T1	T2	*p*-Value
**Laboratory markers**						
Haemoglobin (g/L) ^2^	91 (84–116)	83 (80–88)	<0.001	115 (96–129)	92 (85–98)	<0.001
Leukocytes (×10^9^/L) ^2^	13.8 (9.9–19.6)	10.6 (8.0–16.4)	0.054	11.3 (9.4–14.9)	10.6 (8.5–14.6)	0.627
CRP (mg/L) ^2^	102.8 (7.3–198.0)	193.0 (133.5–290.3)	0.012	38.9 (6.2–109.8)	169.7 (108.9–215.4)	<0.001
PCT (ng/mL) ^2^	1.4 (0.2–14.2)	2.1 (0.7–5.9)	0.035	0.2 (0.1–0.75)	0.6 (0.2–2.15)	0.381
Platelets (10^3^/L) ^2^	129 (86–204)	57 (29–79)	<0.001	189 (128–256)	123 (96–200)	0.040
Total bilirubin (mg/dL) ^2^	0.7 (0.5–1.6)	1.3 (0.7–2.0)	0.013	1.0 (0.5–1.2)	1.0 (0.6–1.5)	0.064
AST (U/L) ^2^	427 (131–668)	200 (62–578)	0.036	113 (55–247)	79 (42–202)	0.135
ALT (U/L) ^2^	154 (72–285)	65 (38–260)	0.010	71 (32–159)	51 (42–87)	0.099
LDH (U/L) ^2^	812 (475–1541)	604 (380–806)	0.039	390 (319–771)	435 (294–547)	0.424
Albumin (g/dL) ^2^	25 (23–30)	22 (21–25)	0.011	30 (26–33)	26 (24–28)	0.038
Creatine Kinase (U/L) ^2^	1223 (278–4471)	553 (172–6031)	0.209	520 (91–1105)	245 (148–1348)	0.903
Myoglobin (ng/mL) ^2^	2183 (462–5091)	561 (294–4054)	0.012	541 (196–1491)	361 (239–891)	0.372
pH ^2^	7.37 (7.30–7.44)	7.41 (7.34–7.47)	0.055	7.36 (7.29–7.45)	7.41 (7.39–7.45)	0.114
Base excess (mmol/L) ^2^	−2.9 (−6.05–1)	1.8 (−3.9–3.3)	0.008	−1.10 (−4.20–0.8)	2.5 (0.6–5.2)	<0.001
Lactate (mmol/L) ^2^	4.1 (1.5–8.4)	1.1 (0.9–4.4)	<0.001	1.5 (1.0–6.3)	1.0 (0.6–1.4)	0.004
**Respiratory parameters**						
PEEP (mbar) ^2^	10 (8–10)	9 (8–11)	0.574	8 (7–10)	8 (7–9)	0.631
pInsp (mbar) ^2^	26 (21–28)	24 (21–28)	0.749	24 (21–28)	23 (19–24)	0.200
Horovitz index ^2^	160 (110.5–206.5)	195 (158.8–234.8)	0.530	249 (174–310)	218 (148–275)	0.469
**Hemodynamic and organ function parameters**						
mAFP flow rate (L/min) ^2^	2 (1.5–2.2)	1.3 (1–2.2)	0.240	2.1 (2.0–2.2)	2.1 (1.4–2.3)	0.131
mAFP P-Level ^2^	4 (3–6)	2 (2–5)	0.390	6 (6–6)	5 (4–6)	0.102
VA-ECMO flow rate (L/min) ^2^	3.0 (2.5–3.4)	2.2 (1.5–2.9)	0.002	3.1 (2.3–3.8)	3.1 (2.7–3.4)	0.833
VIS ^2^	70.0 (24.7–118.6)	12.1 (5.1–89.8)	0.039	16.7 (8.6–56.3)	10.4 (7.2–20.4)	0.701

## Data Availability

The datasets used and/or analyzed during the current study are available from the corresponding author on request.
